# Supplementary material for: Centrifuger: lossless compression of microbial genomes for efficient and accurate metagenomic sequence classification
Source: Genome Biol. 2024 Apr 25;25:106. doi: 10.1186/s13059-024-03244-4 (PMC11046777; doi:10.1186/s13059-024-03244-4)

Table S1. The classification accuracy at various taxonomy ranks in the Mason-generated simulated data Sensitivity=TP/T, Precision=TP/P. The highest values of sensitivity and precision at each rank are bolded.

rank	Matched (TP)	Predicted (P)	Cases (T)	sensitivity	precision	method
strain	231270	241184	1000000	**0.2313**	0.9589	Centrifuger
	229849	441444	1000000	0.2298	0.5207	Centrifuge
	221359	232119	1000000	0.2214	0.9536	Kraken2
	229078	234138	1000000	0.2291	0.9784	Ganon
	173246	173579	1000000	0.1732	**0.9981**	KMCP
species	816975	832477	1000000	**0.8170**	0.9814	Centrifuger
	607293	618127	1000000	0.6073	0.9825	Centrifuge
	607231	626061	1000000	0.6072	0.9699	Kraken2
	680849	690484	1000000	0.6808	0.9860	Ganon
	378273	378309	1000000	0.3783	**0.9999**	KMCP
genus	967470	972537	999521	**0.9679**	0.9948	Centrifuger
	765665	771680	999521	0.7660	0.9922	Centrifuge
	913546	918606	999521	0.9140	0.9945	Kraken2
	935026	938181	999521	0.9355	0.9966	Ganon
	853067	853070	999521	0.8535	**1.0000**	KMCP
family	992539	992922	997389	**0.9951**	0.9996	Centrifuger
	782725	785742	997389	0.7848	0.9962	Centrifuge
	988327	988860	997389	0.9909	0.9995	Kraken2
	991135	991571	997389	0.9937	0.9996	Ganon
	969460	969460	997389	0.9720	**1.0000**	KMCP
order	995976	996205	998781	**0.9972**	0.9998	Centrifuger
	786747	788930	998781	0.7877	0.9972	Centrifuge
	993684	994021	998781	0.9949	0.9997	Kraken2
	995400	995668	998781	0.9966	0.9997	Ganon
	980796	980796	998781	0.9820	**1.0000**	KMCP
class	996386	996466	997481	**0.9989**	0.9999	Centrifuger
	787116	788637	997481	0.7891	0.9981	Centrifuge
	994895	995050	997481	0.9974	0.9998	Kraken2
	996150	996238	997481	0.9987	0.9999	Ganon
	985743	985743	997481	0.9882	**1.0000**	KMCP
phylum	999402	999448	999749	**0.9997**	**1.0000**	Centrifuger
	790843	791705	999749	0.7910	0.9989	Centrifuge
	998390	998475	999749	0.9986	0.9999	Kraken2
	999219	999240	999749	0.9995	**1.0000**	Ganon
	990439	990439	999749	0.9907	**1.0000**	KMCP

Table S2. SRA IDs of the samples used in the SARS-CoV-2 sequence-level classification analysis

(a) PRJNA673096

SRR22712536,SRR22191080,SRR20667346,SRR20666979,SRR21914660,SRR21914666,SRR22191416,SRR
21914609,SRR22191435,SRR21914437,SRR21914640,SRR21915121,SRR21914808,SRR21914667,SRR219
14796,SRR21915275,SRR20666999,SRR22191195,SRR22191062,SRR22191537,SRR22191431,SRR219152
81,SRR21914604,SRR21915073,SRR20666871,SRR22191151,SRR21915094,SRR21914795,SRR22191157,
SRR22191412,SRR21914469,SRR21914723,SRR21914600,SRR20666605,SRR21914699,SRR21914648,SRR
21914809,SRR22191091,SRR22191404,SRR21915019,SRR22191314,SRR21914463,SRR20666983,SRR219
15118,SRR22191539,SRR22191092,SRR22191538,SRR21914807,SRR21914844,SRR21914697,SRR219146
58,SRR21915271,SRR21914671,SRR22191414,SRR21915142,SRR22191059,SRR21915088,SRR22191090,
SRR22191307,SRR20666976,SRR21915100,SRR22712504,SRR21914459,SRR21915077,SRR21914466,SRR
21915141,SRR21914652,SRR20666608,SRR21915130,SRR22191415,SRR22191417,SRR21914627,SRR219
15072,SRR21914624,SRR21914506,SRR20666998,SRR21914451,SRR21915138,SRR22191533,SRR206666
03,SRR21915277,SRR21914436,SRR22191536,SRR20666992,SRR20666971,SRR21914608,SRR21914606,
SRR22191298,SRR20666960,SRR20666975,SRR21914619,SRR21915139,SRR21914840,SRR21914651,SRR
21915129,SRR20666978,SRR21914623,SRR20666950,SRR20666996,SRR20666606

(b) PRJEB40277

ERR7616152,ERR6752462,ERR10806662,ERR6894671,ERR7280780,ERR6087467,ERR6894729,ERR10808
805,ERR10808806,ERR9877510,ERR6281163,ERR6281100,ERR6894527,ERR7251916,ERR11175028,ERR7
251827,ERR8264476,ERR6359553,ERR7251918,ERR6535794,ERR7251899,ERR6281107,ERR6535801,ERR
6132778,ERR6535803,ERR7251940,ERR6752499,ERR7251964,ERR6894743,ERR6535797,ERR6894607,ER
R6894175,ERR9958460,ERR7251800,ERR6394400,ERR6099153,ERR6281092,ERR6281198,ERR7251981,E
RR7251913,ERR7251924,ERR6894634,ERR6099131,ERR6894673,ERR7365342,ERR6281017,ERR6929099,
ERR6281252,ERR8137994,ERR9877517,ERR6132756,ERR6496854,ERR8264549,ERR7365341,ERR907734
9,ERR6616458,ERR6132760,ERR9877522,ERR6394520,ERR6281232,ERR7334708,ERR7365347,ERR68946
90,ERR6099059,ERR6281172,ERR6894172,ERR6496849,ERR6099128,ERR6099029,ERR6087566,ERR6894
752,ERR7251502,ERR7365339,ERR7365340,ERR7365345,ERR6281131,ERR7616166,ERR6894742,ERR736
5352,ERR10806592,ERR6394399,ERR6752497,ERR7251908,ERR6894718,ERR7365343,ERR7251898,ERR6
894721,ERR7251922,ERR7251979,ERR7251949,ERR6894668,ERR7251894,ERR11274576,ERR11267813,E
RR11267798,ERR11267833,ERR11274577,ERR11274575,ERR11274574,ERR11274573

Table S3. SRA IDs of the samples used in the bacterial WGS classification evaluations

(a) Species-in samples

SRR22307691,SRR21536800,ERR10440122,SRR22973785,SRR24915652,ERR10435774,SRR23291419,SRR21232130,ERR10422207,SRR21391477,SRR23269275,ERR11049760,ERR10891225,SRR22862827,SRR25393410,ERR10361812,SRR21472570,SRR23344037,SRR22051901,SRR25322644,ERR11209666,SRR23937127,ERR10702869,SRR23080377,SRR21472777,SRR24876935,SRR24660299,SRR23320234,SRR22839128,SRR24894189,SRR24326338,ERR10890411,SRR21160816,SRR25361444,SRR22826819,SRR24988115,SRR24467445,SRR23274612,SRR23355009,SRR24884283,SRR21684637,SRR23425431,ERR10074672,SRR21541429,SRR24969135,SRR24743436,SRR22980637,SRR24890545,ERR10161818,SRR21733551,SRR23697489,SRR21472699,SRR21472879,SRR23190414,ERR10759922,SRR23373793,SRR22536093,SRR21472820,SRR22062739,SRR24742604,SRR24326226,ERR10305174,SRR25072685,SRR21434628,SRR23384699,SRR25145571,ERR7425868,ERR10300512,SRR22096772,SRR19070098,SRR22550467,SRR23885914,ERR10359856,SRR25206002,SRR24969169,SRR24958400,SRR23053888,SRR24984115,SRR23912828,SRR22981646,SRR22981039,SRR23133853,SRR23033313,SRR21538116,ERR10390746,SRR21745898,SRR22980906,SRR24446366,SRR21595977,SRR22207084,SRR23727810,SRR24967447,SRR25301688,SRR22980750,SRR21002050,SRR23821289,SRR21221814,SRR21472983,SRR25209441,SRR24748826

(b) Species-not-in samples

SRR23587921,SRR25145326,SRR22426788,SRR22223269,SRR19184703,SRR25145603,SRR23515135,ERR10233522,SRR23109617,SRR25050399,SRR23199814,SRR21922626,SRR25249328,SRR22980881,SRR22533712,SRR22981063,SRR23719805,SRR25161135,SRR22980831,SRR24757566,ERR10474896,SRR23216202,ERR10474912,SRR23316565,SRR21922640,ERR10474890,ERR10502705,SRR24651275,SRR21922795,SRR24887169,ERR10784662,SRR25145641,ERR10474901,SRR23515134,SRR23384168,ERR10089233,SRR22980720,SRR24969114,SRR24969137,ERR10474903,ERR10474919,SRR21337366,SRR22904081,SRR24887474,SRR24173321,SRR24878659,SRR21922773,SRR24300470,SRR24736436,SRR24886697,SRR21922762,ERR10474915,SRR22751338,SRR24887463,ERR10474911,ERR10474876,SRR22980838,SRR21922727,SRR25145544,SRR24969109,SRR25246370,SRR24886967,SRR25145604,SRR22751340,SRR22395562,SRR25145511,SRR25302806,ERR10474905,SRR24886695,SRR21922827,ERR10430216,SRR24204389,SRR21537771,ERR10897550,SRR22285489,ERR10431703,SRR24651314,SRR23852874,SRR23308057,SRR24902343,SRR24969179,SRR24886694,SRR23754999,SRR23291491,SRR21658418,ERR10229495,SRR21617132,SRR23096974,SRR21922778,SRR21315072,SRR23285933,SRR24300501,SRR23436780,SRR23313049,SRR25145423,SRR24736439,SRR22443693,SRR25145435,SRR23116489, SRR23157132

Table S4. Running commands for the classifiers used in the evaluations

Method	Command ($t: threads; $index: path to index; $r1, $r2: read files)
Centrifuger v1.0.1	centrifuger -t $t -x $index -1 $r1 -2 $r2 > result.out<br><br>Long read: centrifuger -t $t -x $index -u $r1 > result.out
Centrifuge v1.0.4	centrifuge-class -p $t -x $index -1 $r1 -2 $r2 --no-abundance > result.out
Kraken2 v2.1.3	kraken2 --paired –db $index --threads $t $r1 $r2 > result.out<br><br>Long read: kraken2–db $index --threads $t $r1 > result.out
Ganon v2.0.0	ganon classify --db-prefix $index --output-prefix result -t $t -b --output-one --multiple-matches lca --paired-read $r1 $r2<br><br>Long read: ganon classify --db-prefix $index --output-prefix result -t $t --output-one --multiple-matches lca -s $r1 --rel-cutoff 0.12 --rel-filter 0.9
KMCP v0.9.4	kmcp search -d $index -o result.out --threads $t -w -1 $r1 -2 $r2
MetaMaps v633d2e0	metamaps mapDirectly -t $t --all -r $index/DB.fa -o map_results<br>metamaps classify --mappings map_results -t $t --DB $index -q $r1
Taxor v0.1.0	taxor search --index-file $index --output-file result.out --threads $t --query-file $r1

Fig. S1. Space usage of the wavelet tree, RLBWT, hybrid run-length compression and RBBWT when adding genomes with (A) the species *Escherichia fergusonii* (taxonomy ID 564) and (B) the genus *Legionella* (taxonomy ID 445)

(A)

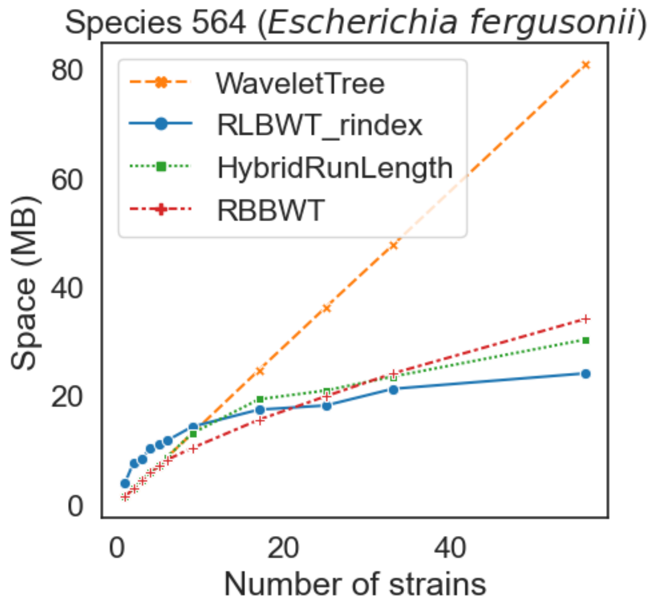

(B)

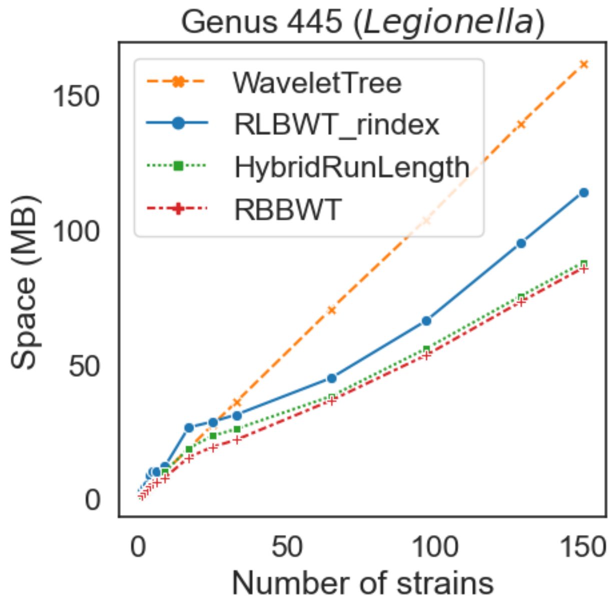

Fig. S2. Space usage of the wavelet tree, RLBWT, hybrid run-length compression and RBBWT when adding genomes with species *Chalmydia trachomatis* (taxonomy ID 813) and the genus *Chalmydia* (taxonomy ID 810)

(A) Space usages for representing the species *Chalmydia trachomatis*. Left: absolute space usage in megabytes (MB). Right: bits used to represent one base pair (bp) when the average run length of the BWT sequence (n/r) increases.

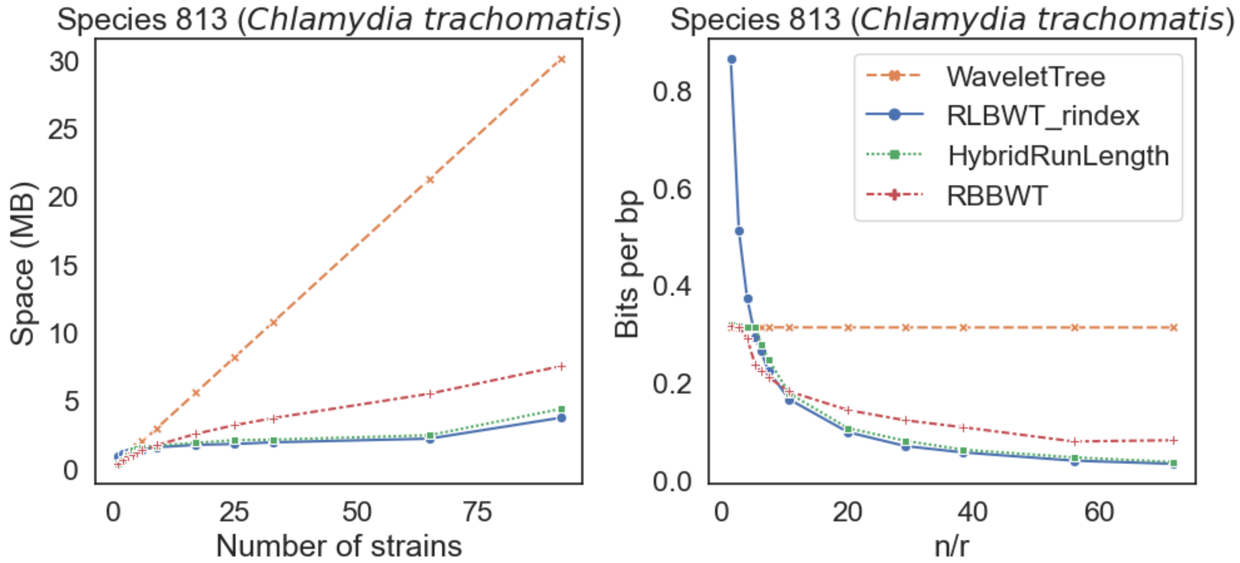

(B) Space usages for representing the genus *Chalmydia*. Left: absolute space usage in megabytes (MB). Right: bits used to represent one base pair (bp) when the average run length of the BWT sequence (n/r) increases.

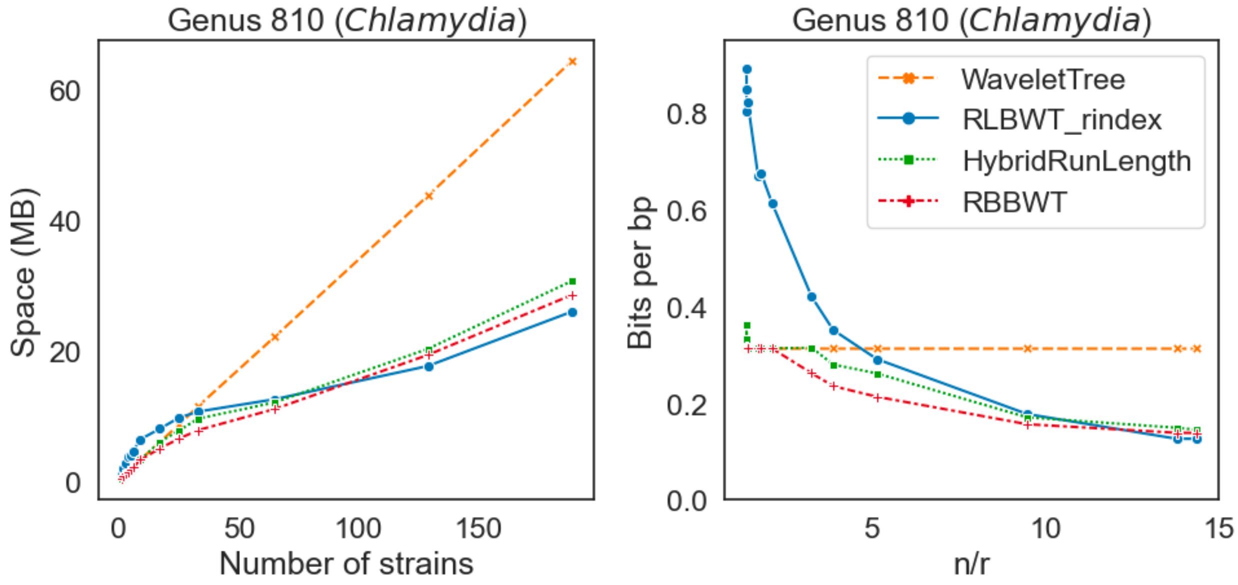

Fig. S3. Sensitivity (left) and precision (right) of Centrifuger, Centrifuge, Kraken2, Ganon, and KMCP on the simulated data generated from June 2023 RefSeq prokaryotic genomes using ART

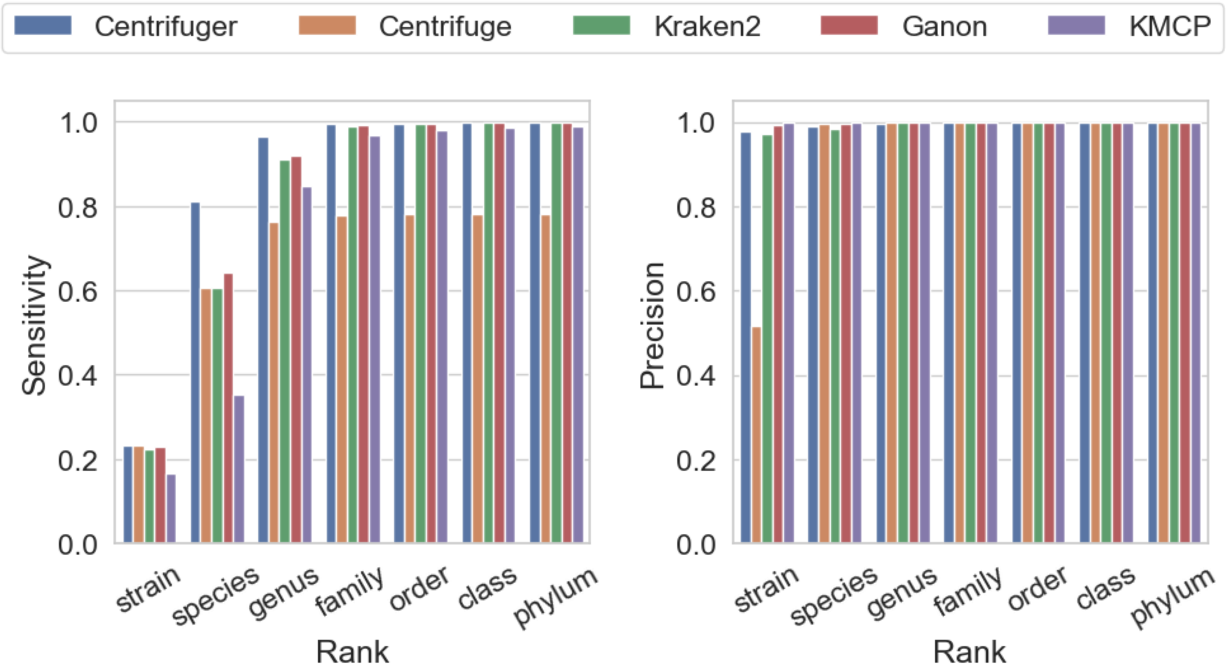

Fig. S4. Performance of Centrifuger, Centrifuge, Kraken2, Ganon, and KMCP on the simulated data when classifying against a trimmed database that has one genome per genus and does not contain the true origins of the reads

(A) Sensitivity (left) and precision (right)

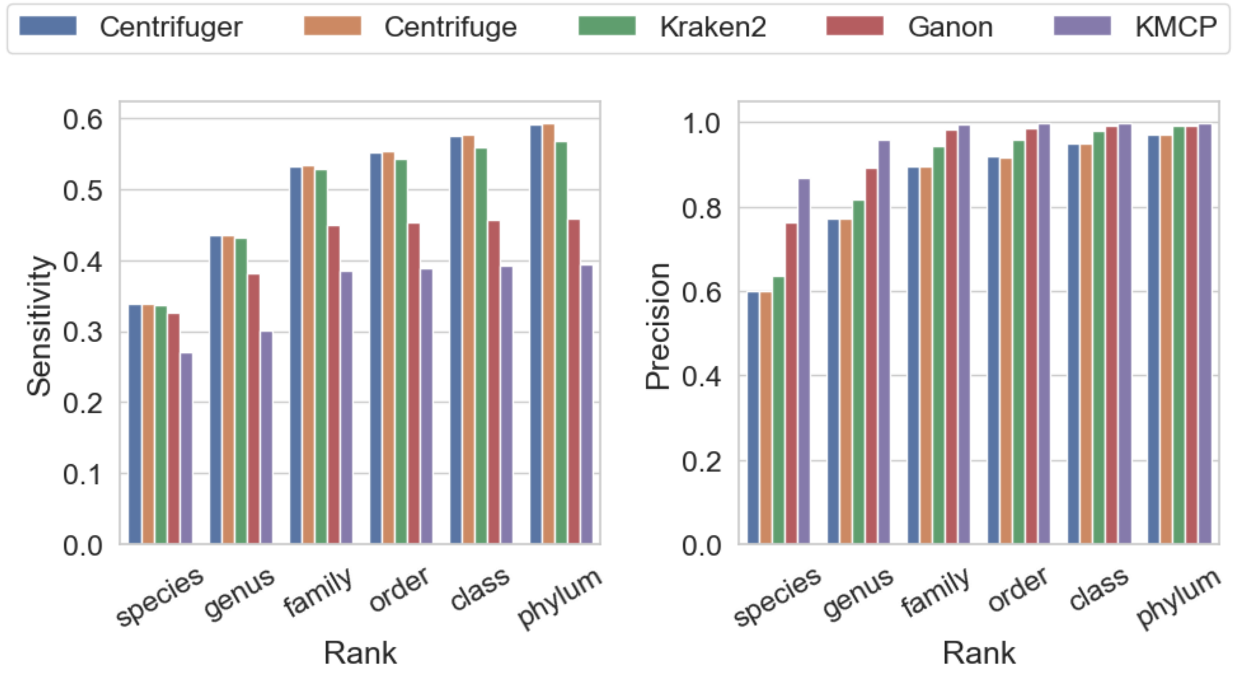

(B) F1 score (2*sensitivity*precision/(sensitivity + precision))

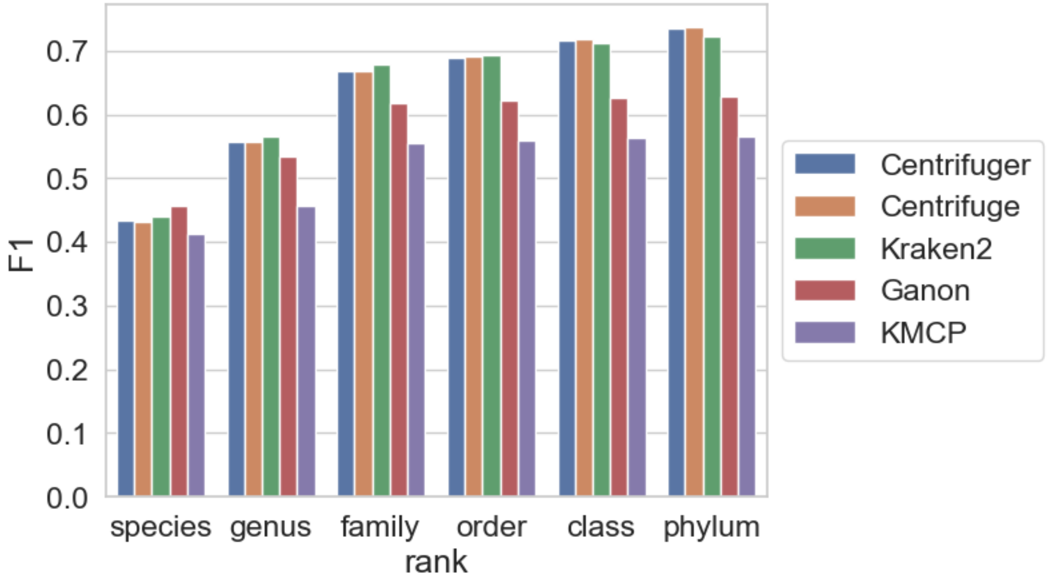

Fig. S5. F1 scores of Centrifuger, Centrifuge, Kraken2, Ganon, and KMCP at various taxonomy ranks on the 10 simulated data sets from CAMI2

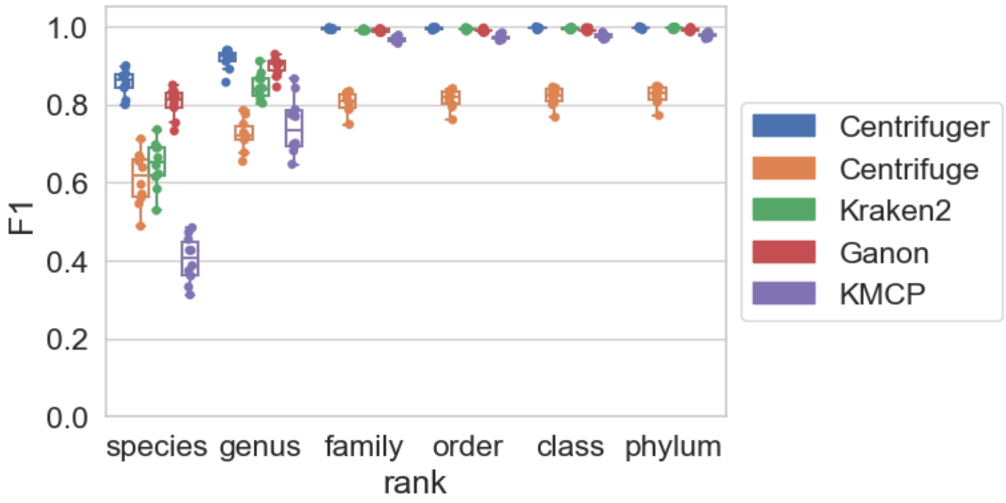

Fig. S6. F1 scores of Centrifuger, Centrifuge, Kraken2, Ganon and KMCP on bacterial WGS data sets, where the species of the bacteria are not in the database but their genera are present in the database

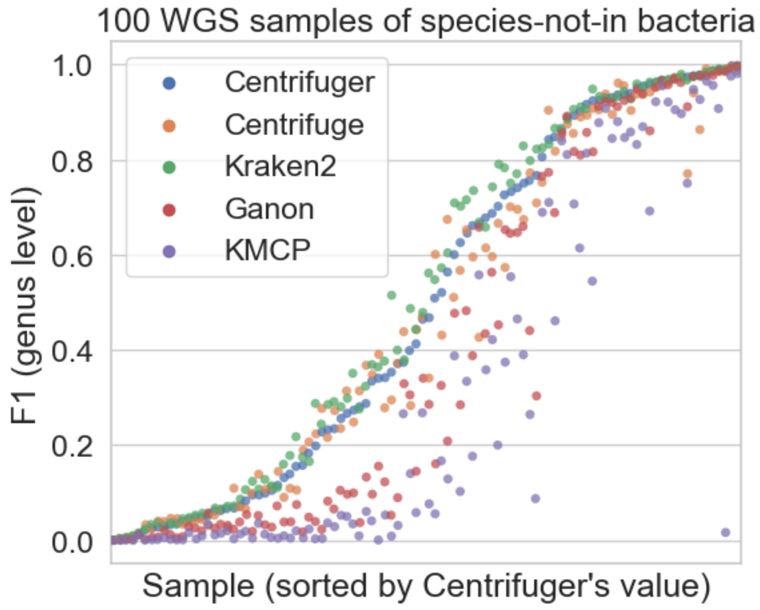

Fig. S7. Cluster heatmaps for read fractions in the SARS-CoV-2 sequence-level analysis
The rows are SARS-CoV-2 variants present in the RefSeq and GenBank, and the columns are the Oxford Nanopore WGS samples.
(A) Raw values of read factions for each sequence ID from Centrifuger

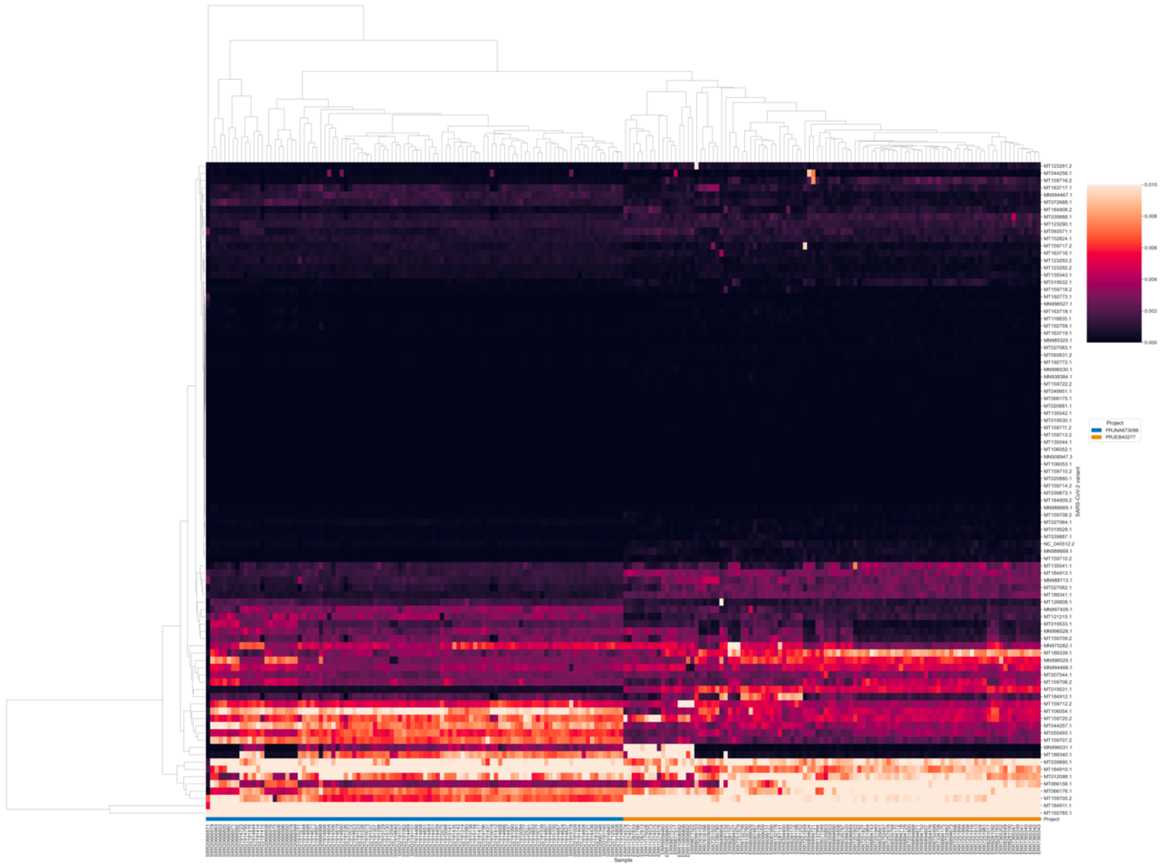

(B) Z-score transformed read fraction for each sequence ID from Centrifuge

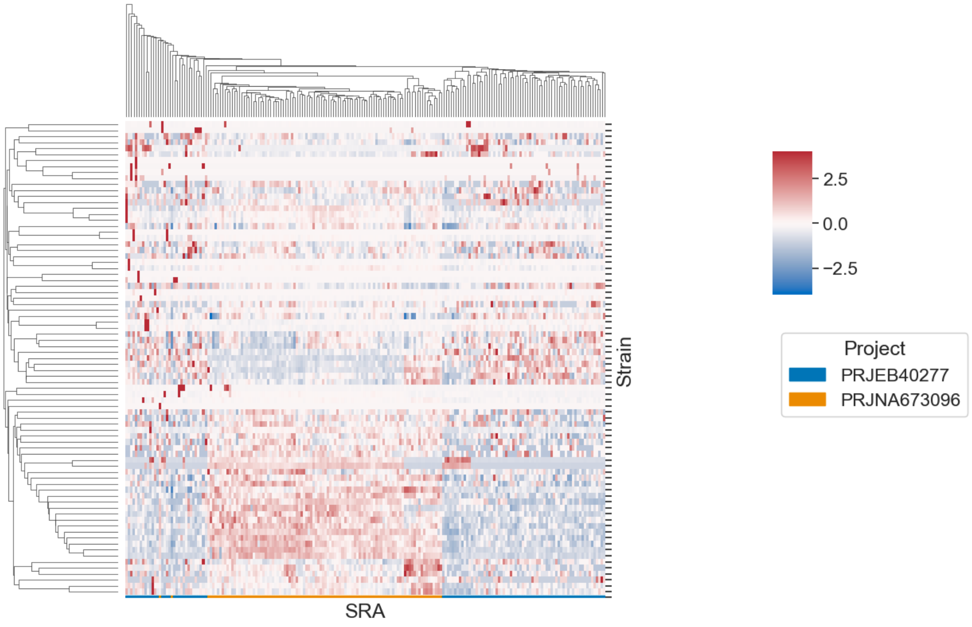

Fig. S8. Sensitivity, precision and F1 score of Centrifuger and Ganon using LCA and reassignment based on taxa quantification at various taxonomy levels

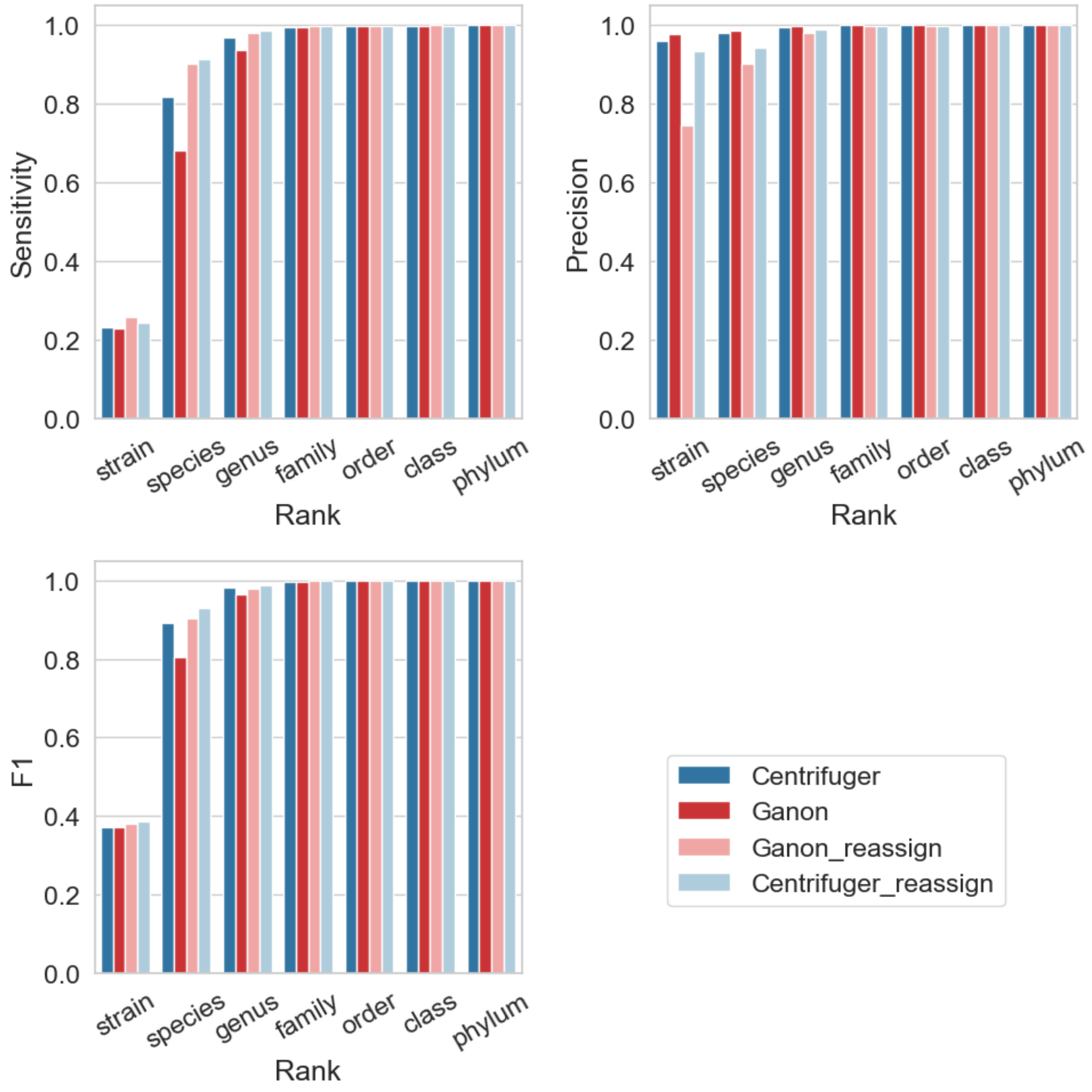

Supplement: Supplementary file 1 — Additional file 1: Table S1. The classification accuracy at various taxonomy ranks in the Mason-generated simulated data Table S2. SRA IDs of the samples used in the SARS-CoV-2 sequence-level classification analysis. Table S3. SRA IDs of the samples used in the bacterial WGS classification evaluations. Table S4. Running commands for the classifiers used in the evaluations. Fig. S1. Space usage of the wavelet tree, RLBWT, hybrid run-length compression and RBBWT when adding genomes with the species Escherichia fergusonii (taxonomy ID 564) and the genus Legionella (taxonomy ID 445) Fig. S2. Space usage of the wavelet tree, RLBWT, hybrid run-length compression and RBBWT when adding genomes with species Chalmydia trachomatis (taxonomy ID 813) and the genus Chalmydia (taxonomy ID 810). Fig. S3. Sensitivity and precision of Centrifuger, Centrifuge, Kraken2, Ganon, and KMCP on the simulated data generated from June 2023 RefSeq prokaryotic genomes using ART. Fig. S4. Performance of Centrifuger, Centrifuge, Kraken2, Ganon, and KMCP on the simulated data when classifying against a trimmed database that has one genome per genus and does not contain the true origins of the reads. Fig. S5. F1 scores of Centrifuger, Centrifuge, Kraken2, Ganon, and KMCP at various taxonomy ranks on the 10 simulated data sets from CAMI2. Fig. S6. F1 scores of Centrifuger, Centrifuge, Kraken2, Ganon and KMCP on bacterial WGS data sets, where the species of the bacteria are not in the database but their genera are present in the database. Fig. S7. Cluster heatmaps for read fractions in the SARS-CoV-2 sequence-level analysis. Fig. S8. Sensitivity, precision and F1 score of Centrifuger and Ganon using LCA and reassignment based on taxa quantification at various taxonomy levels. [file 13059_2024_3244_MOESM1_ESM.pdf]
